# Supplementary material for: Mechanisms governing the pioneering and redistribution capabilities of the non-classical pioneer PU.1
Source: Nat Commun. 2020 Jan 21;11:402. doi: 10.1038/s41467-019-13960-2 (PMC6972792; doi:10.1038/s41467-019-13960-2)
Supplement: Supplementary file 7 — Source data [file 41467_2019_13960_MOESM7_ESM.zip › Source_Data/Figure4/Figure4B_MotifScanOutput/knownResults.html]

/misc/data/analysis/project\_PU1/CTV1/motifs/basic/PU1\_Kmeans14.all.redhomer - Homer Known Motif Enrichment Results


# Homer Known Motif Enrichment Results (/misc/data/analysis/project\_PU1/CTV1/motifs/basic/PU1\_Kmeans14.all.redhomer)

Homer *de novo* Motif Results  
Gene Ontology Enrichment Results  
Known Motif Enrichment Results (txt file)  
Total Target Sequences = 31883, Total Background Sequences = 31159

|  |  |  |  |  |  |  |  |  |  |  |  |
| --- | --- | --- | --- | --- | --- | --- | --- | --- | --- | --- | --- |
| Rank | Motif | Name | P-value | log P-pvalue | q-value (Benjamini) | # Target Sequences with Motif | % of Targets Sequences with Motif | # Background Sequences with Motif | % of Background Sequences with Motif | Motif File | SVG |
| 1 | A T G C C G T A A T G C A C G T A C G T A G T C A G T C A G C T A G T C G C A T G C A T G C A T G A C T A G C T | 1-CACTTCCTCWTTTN,BestGuess:SpiB(ETS)/OCILY3-SPIB-ChIP-Seq(GSE56857)/Homer(0.966) | 1e-4112 | -9.471e+03 | 0.0000 | 15237.0 | 47.79% | 1000.2 | 3.21% | motif file (matrix) | svg |
| 2 | A T C G G C A T C T G A T C G A A G T C A T G C C T G A T G A C C T G A C T G A | 4-NWAACCACAD,BestGuess:RUNX(Runt)/HPC7-Runx1-ChIP-Seq(GSE22178)/Homer(0.973) | 1e-145 | -3.348e+02 | 0.0000 | 5595.0 | 17.55% | 3258.8 | 10.47% | motif file (matrix) | svg |
| 3 | G T C A T C G A A C T G A T C G G C T A C G T A A C T G A C G T C A T G G T C A C T G A C G T A | 6-NAKGAAGTGAAA,BestGuess:PU.1:IRF8(ETS:IRF)/pDC-Irf8-ChIP-Seq(GSE66899)/Homer(0.885) | 1e-88 | -2.027e+02 | 0.0000 | 674.0 | 2.11% | 128.3 | 0.41% | motif file (matrix) | svg |
| 4 | T G C A T G C A G T A C G T A C G T C A G T A C G T C A G C A T A T G C G A T C A G C T A T G C G A C T | 7-AACCACATCCTCT,BestGuess:ETS:RUNX(ETS,Runt)/Jurkat-RUNX1-ChIP-Seq(GSE17954)/Homer(0.882) | 1e-75 | -1.746e+02 | 0.0000 | 767.0 | 2.41% | 202.6 | 0.65% | motif file (matrix) | svg |
| 5 | C A T G G A T C A G T C C T G A T A G C G A C T C T G A A C T G C T G A A C T G A C T G C A T G G A T C T C G A | 4-GCCACTAGAKGGCA,BestGuess:CTCF(Zf)/CD4+-CTCF-ChIP-Seq(Barski\_et\_al.)/Homer(0.927) | 1e-56 | -1.304e+02 | 0.0000 | 475.0 | 1.49% | 102.4 | 0.33% | motif file (matrix) | svg |
| 6 | C G T A C G T A A G T C A G T C A C T G G C T A G T C A | 9-AACCGHA,BestGuess:MYB81(MYB)/col-MYB81-DAP-Seq(GSE60143)/Homer(0.939) | 1e-37 | -8.642e+01 | 0.0000 | 13890.0 | 43.57% | 11991.6 | 38.52% | motif file (matrix) | svg |
| 7 | T G C A C A G T A T C G G T C A C A T G A G C T T A G C T C G A A G C T | 8-ATGAGTCAT,BestGuess:JDP2/MA0655.1/Jaspar(0.965) | 1e-33 | -7.813e+01 | 0.0000 | 1951.0 | 6.12% | 1242.5 | 3.99% | motif file (matrix) | svg |
| 8 | C T G A A G T C C G T A A C T G A G T C C G A T A C T G | 15-ACAGCTG,BestGuess:MyoG(bHLH)/C2C12-MyoG-ChIP-Seq(GSE36024)/Homer(0.971) | 1e-20 | -4.742e+01 | 0.0000 | 9461.0 | 29.67% | 8190.7 | 26.31% | motif file (matrix) | svg |
| 9 | A G T C A T G C C A G T A C G T G C A T A C T G G C T A G C A T T A C G A G C T | 8-YCTTTGATST,BestGuess:TCF7L2/MA0523.1/Jaspar(0.918) | 1e-12 | -2.823e+01 | 0.0000 | 1950.0 | 6.12% | 1502.9 | 4.83% | motif file (matrix) | svg |
| 10 | C A G T C T A G G A C T T G C A T G C A C T G A G T A C T C G A | 7-TGTAAACA,BestGuess:Foxo3(Forkhead)/U2OS-Foxo3-ChIP-Seq(E-MTAB-2701)/Homer(0.960) | 1e-9 | -2.195e+01 | 0.0000 | 9066.0 | 28.44% | 8167.2 | 26.23% | motif file (matrix) | svg |
| 11 | G T A C A G T C G C A T C G T A A C G T A G T C C T G A T C A G | 6-CBTATCRV,BestGuess:GLN3/MA0307.1/Jaspar(0.910) | 1e-9 | -2.127e+01 | 0.0000 | 3907.0 | 12.25% | 3333.5 | 10.71% | motif file (matrix) | svg |
| 12 | A C G T C A G T C G A T C A T G A G T C C T G A C G A T G C T A C G T A | 16-TTTGCATAA,BestGuess:Oct4(POU,Homeobox)/mES-Oct4-ChIP-Seq(GSE11431)/Homer(0.895) | 1e-7 | -1.642e+01 | 0.0000 | 2890.0 | 9.06% | 2458.4 | 7.90% | motif file (matrix) | svg |
| 13 | C T G A T C A G A G T C A G T C C G T A G T C A G A C T T A G C T C G A T C A G T G C A C T A G | 2-AGCCAATCRGVG,BestGuess:NFY(CCAAT)/Promoter/Homer(0.963) | 1e-4 | -9.239e+00 | 0.0001 | 1098.0 | 3.44% | 909.5 | 2.92% | motif file (matrix) | svg |
